# Determinants and underlying causes of frequent attendance in midwife-led care: an exploratory cross-sectional study

**DOI:** 10.1186/s12884-019-2316-5

**Published:** 2019-06-17

**Authors:** Janneke T. Gitsels – van der Wal, Lisanne A. Gitsels, Angelo Hooker, Britte van Weert, Linda Martin, Esther I. Feijen – de Jong

**Affiliations:** 1 Amsterdam UMC, Vrije Universiteit Amsterdam, Midwifery Science, AVAG, Amsterdam Public Health research institute, De Boelelaan 1117, Amsterdam, The Netherlands; 2 ESRC funded Business and Local Government Data Research Centre (BLG DRC), School of Computing Sciences, University of East Anglia, Norwich Research Park, Norwich, NR4 7TJ England, UK; 3 Department of Obstetrics and Gynaecology, Amsterdam UMC, Vrije Universiteit Amsterdam, De Boelelaan 1117, Amsterdam, Netherlands; 4 Department of Obstetrics and Gynecology, Zaans Medical Center, Koningin Julianaplein 58, Zaandam, Netherlands; 5 Department of General Practice & Elderly Care Medicine, University of Groningen, University Medical Center Groningen, PO Box 30001, 9700 RB Groningen, the Netherlands

**Keywords:** Midwifery, Prenatal care, Patient acceptance of health care, Social determinants of health, Sexual violence, Psychosocial deprivation

## Abstract

**Background:**

An adequate number of prenatal consultations is beneficial to the health of the mother and fetus. Guidelines recommend an average of 5–14 consultations. Daily practice, however, shows that some women attend the midwifery practice more frequently. This study examined factors associated with frequent attendance in midwifery-led care.

**Methods:**

We conducted a cross-sectional study in a large midwifery practice in the Netherlands among low-risk women who started prenatal care in 2015 and 2016. Based on Andersen’s behavioral model, we collected data on potential determinants from the digital midwifery’s practice database. Prenatal healthcare utilization was measured by a revised version of the Kotelchuck Index, which measures a combination of care entry and numbers of visits. Logistic regression models were fitted to estimate the likelihood of frequent attendance compared to the recommended number of visits, adjusted for all relevant factors. Separate models were fitted on the non-referred and the referred group of obstetric-led care, as referral was found to be an effect modifier.

**Results:**

The prevalence of frequent attendance was 23% (243/1053), mainly caused by worries and/or vague complaints (44%; 106/243). Among non-referred women, 53% (560/1053), frequent attendance was associated with consultation with an obstetrician (OR = 3.99 (2.35–6.77)) and exposure to sexual violence (OR = 2.17 (1.11–4.24)). Among the referred participants, 47% (493/1053), frequent attendance was associated with a consultation with an obstetrician (OR = 2.75 (1.66–4.57)), psychosocial problems in the past or present (OR = 1.85 (1.02–3.35) or OR = 2.99 (1.43–6.25)), overweight (OR = 1.88 (1.09–3.24)), and deprived area (OR = 0.50 (0.27–0.92)).

**Conclusion:**

Our exploratory study indicates that the determinants of frequent attendance in midwifery-led care differs between non-referred and referred women. Underlying causes for frequent attendance was mainly because of non-medical reasons. Implication for practice**:** A trustful midwife-client relationship is known to be needed for clients such as frequent attenders to share more detailed, personal stories in case of vague complaints or worries, which is necessary to identify their implicit needs.

## Background

In 2016 the World Health Organisation advised at least eight prenatal visits during pregnancy to decrease perinatal mortality and to increase positive experiences of pregnant women [1]. The National Institute for Health and Care Excellence (NICE) advises three additional visits for nulliparous women [2]. Despite these recommendations, the number of face-to-face visits varies between five and fourteen worldwide [1–4]. The current prenatal care schedule of low-risk pregnant women in the Netherlands comprises an average of 14 visits in pregnancy, the highest number of visits for low-risk women worldwide [5]. In addition, the Royal Dutch Organisation of Midwives (KNOV – Koninklijke Nederlandse Organisatie van Verloskundigen) recommends adjusting the number of prenatal visits to the medical care, needs, and preferences of the woman. More visits could be initiated by the care provider as well as by the caretaker [5–7].

Frequent attendance, defined as patients receiving significantly more visits than recommended, has hardly been studied in midwifery-led care whereas it is a recognized problem in general practice and hospitals. For instance, 10% of patients account for approximately 40% of encounters to general practices [8, 9]. Frequent attendance has been associated with psychosocial problems such as anxiety, high perceived stress, low self-esteem and poor self-regulation [8, 9]. Women are more often frequent attenders than men [10]. Feijen-de Jong et al. investigated prenatal health care utilization in primary midwifery care in a multicentre prospective dynamic cohort study in the Netherlands and showed that 66% of the participants had received the recommended number of visits, 21% fewer, and 13% more [3]. A Swedish study showed that 25% received the recommended number of visits, 17% fewer and 57% more [7].

Previous studies have found a positive association between frequent attendance and preterm birth, low birth-weight, perinatal mortality, lower Apgar scores, induction of labour and caesarean section [11–14]. Due to the increasing number of women seem to be having more visits than recommended, resulting in increased workload of midwives and their clinics without apparent improvement of outcomes, it is essential to understand why some women require more visits than others; understanding this phenomenon can contribute to the improvement of the healthcare organization, planning and execution of care and most importantly, might improve perinatal outcomes. The aim of this study was therefore to examine the prevalence, determinants, and underlying causes of frequent attendance in midwife-led care, and whether these differ from those who are referred to an obstetrician. We used Andersen’s behavioral model of healthcare use as a guiding framework to categorize the determinants of health care utilization. This model suggests that the use of health care depends on predisposing, enabling, need and health behavior factors [15].

### Dutch healthcare system

The care for pregnant women in the Netherlands is organized as follows: low-risk pregnant women receive prenatal, natal and postnatal care from a midwife, high-risk women receive care from an obstetrician while women with intermediate risk received joint care. In case of a change in the medical condition of a low-risk woman, an obstetrician is consulted and in case of a determined medical condition, the woman is referred to an obstetrician, who will provide care during the remaining pregnancy period and birth. Otherwise, the woman is referred back to the midwife.

Women with psychological problems are often referred to the Psychiatrist-Obstetrician-Pediatrician-clinic, an outpatient setting where pregnant women are evaluated by obstetricians, pediatricians, and psychologists and receive advice from this multi-disciplinary team. For the clarity of this paper, the word ‘visit’ is used related to midwifery-led care, the word ‘consultation’ is used related to consultation of an obstetricians and other specialists in hospitals.

## Methods

### Study design

This exploratory cross-sectional study was conducted in a large midwifery practice, located in a medium-sized city near Amsterdam, the Netherlands. The practice was organized in two teams, each consisting of four midwives. On the 25th of January 2018, the Medical Ethics Committee of the VU University Medical Center (ref. 2018.019) approved this study to collect and analyse retrospective data of January 2015 to January 2017. Women gave permission to use their anonymous data, their verbal consent was obtained during the intake and noted in their files while they watched; the beforementioned Medical Ethics Committee approved this procedure of obtaining consent.

### Participants and measurement tool

Eligible participants were pregnant women at the specified midwifery practice during the study period with an ongoing pregnancy. Women with a miscarriage and women who only received postnatal care were excluded. Included participants were classified based on the level of health care utilization according to the Kotelchuck Index-Revised, see Appendix [3, 12].

In brief, the Kotelchuck Index-Revised, a validated tool for measuring the level of care utilization, is based on the number of face-to-face visits with the midwife, adjusted for gestation and onset of care and aligned with the number of recommended face-to-face prenatal visits by the KNOV. Utilization of care was defined as inadequate (less than 50% of the recommended visits), intermediate (50–79%), adequate (80–109%) and adequate plus (110% or more) [3, 12]. In this study, women with adequate plus utilization of care were considered to be frequent attendance (cases) and were compared to women with the adequate utilization of care (controls). Women with the intermediate or inadequate utilization of prenatal care were excluded from the study because these groups were irrelevant in view of the goal of our study.

### Data collection

The digital midwifery database was searched to identify eligible participants. Two researchers (JG and BW) extracted data from the database, which were anonymized and de-identified. In this database, the medical charts of all pregnant women guided by this midwifery practice are included. The chart includes medical care records of pregnancies, demographic characteristics, lifestyle and medical information of the pregnant woman. Possible reasons for frequent attendance were identified based on the additional information in the charts of frequent attenders documented by the midwife.

Based on the Andersen’s model, several variables concerning predisposing, enabling, need and health behavior factors were considered as potential determinants of frequent attendance [3, 15]. In this study, *Predisposing* variables consisted of sociodemographic factors such as age, ethnicity (Dutch, western non-Dutch, non-western non-Dutch), marital status (relationship yes/no), occupation (yes/no), and level of education. The level of education was subdivided into low (primary school and some vocational training), intermediate (secondary school and completed vocational training), and high (college and/or university). *Enabling* variables included deprivation (yes/no), year of the start of prenatal care (2015/2016), and team (team 1 or team 2); both teams consisted of four midwives. *Needs* variables consisted of perceived and evaluated needs. Perceived needs in this study were telephone consultations initiated by clients (numbers), psychosocial problems (no/yes in the past, yes at present), and sexual violence (no/yes). Evaluated needs included parity (nulliparous or multiparous), the start of pregnancy (spontaneous or assisted), duration of getting pregnant (in months), reasons for frequent attendance, attendance to health care professionals other than the midwife, the number of ultrasound examinations, and prescribed drugs. Reasons for frequent attendance were defined based on the information in clients’ dossiers. Consultation of other healthcare professionals was subdivided in visits with a general practitioner, a medical specialist e.g. an obstetrician or an internist, or a visit to the Psychiatrist-Obstetrician-Pediatrician-clinic (POP). *Health behavior* variables consisted of smoking (no/yes), alcohol consumption (no/yes), intake of folic acid (no/yes), and body mass index before pregnancy (BMI). We classified BMI according to the World Health Organization Classification of adults: underweight and normal weight, overweight, and obesity [16].

### Analyses

Descriptive analysis included contingency tables to summarize the study population. Categorical data were reported as absolute numbers and percentages. Normally distributed continuous variables were reported as means with standard deviations and non-normally distributed continuous variables as medians with interquartile ranges.

Logistic regression models were fitted to estimate the unadjusted and adjusted effects of frequent attendance associated with the variables described above. Variables with a prevalence of less than 5% were excluded from the regression analysis. The team was included in the models as a fixed effect because the two teams provided care to an equal number of participants. Age was re-coded to be centered around the mean to have a meaningful baseline. Backward elimination was performed to obtain the leanest model possible, where each time, the variable with the largest *p*-value was removed from the model until all variables were significant (*p* < .05). The model assumptions were checked and the final model was assessed on overall performance (Nagelkerke’s R2) and discrimination (specificity, sensitivity, and overall accuracy).

Referral to obstetrician was considered an effect modifier of prenatal healthcare utilization.3 Therefore, additional analyses were performed comparing referred and non-referred pregnant women with χ2 tests for categorical variables and separate logistic models were fitted on the subsets of non-referred and referred participants. All analyses were executed using SPSS (version 24).

## Results

**Fig. 1:**
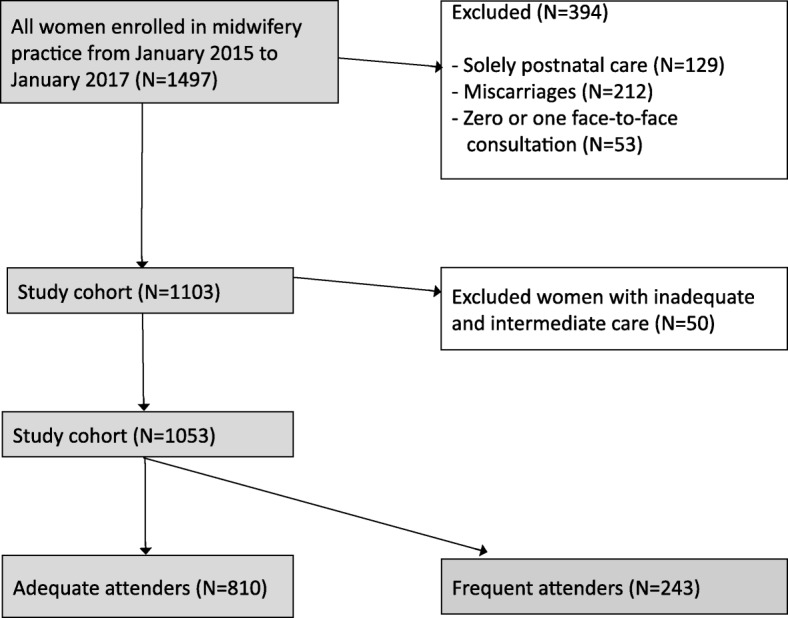
Eligible study population. The flow chart of the eligible study population

Between January 2015 and January 2017, 1497 women received prenatal and/or postnatal care by the midwifery practice. The flow chart of the eligible study population is shown in Fig. 1. The final study population consisted of 1053 women: 810 (77%) received adequate healthcare, and 243 (23%) received adequate plus health care (frequent attendance).

### Results of the complete study group

The characteristics of the study population are shown in Table 1, column 2. The prevalence of frequent attendance in 2015 and 2016 were 19 and 24%, respectively. In our cohort, the average age was 29.2 years, 48% of the women were primipara was 48, and 53% had a non-Dutch background. Of all women, 96 (9%) participants reported to use prescribed drugs, mainly Selective Serotonin Reuptake Inhibitors and thyroid medication.

**Table 1:** Various characteristics of women with adequate plus or adequate use of prenatal care, split by referrals to an obstetrician in the study practice (*N* = 1053)

	Study population *N* = 1053 (%)	Non-referred Adequate plus *N* = 110 (10.4%)	Non-referred Adequate *N* = 450 (42.7%)	Referred Adequate plus *N* = 133 (12.6%)	Referred Adequate *N* = 360 (34.2%)
Healthcare utilization					
Adequate plus (cases)	243 (23.1)				
Adequate (controls)	810 (76.9)				
Referrals					
Non-referred	560 (53.2)				
Referred	493 (46.8)				
Consultations face-to-face					
Mean (sd)	10.8 (4.2)	15.5 (3.6)	11.8 (2.7)	12.5 (4.2)	7.7 (3.6)
Predisposing variables					
Age in years					
Mean (sd)	29.2 (4.2)	28.2 (5.0)	28.7 (4.7)	29.3 (5.1)	30.1 (4.9)
Level of Education^a^					
Low	121 (11.9)	8 (7.5)	49 (11.2)	18 (14.3)	46 (13.1)
Middle (intermediate)	479 (47.0)	55 (51.4)	203 (46.6)	58 (46.0)	163 (46.6)
High	419 (41.1)	44 (41.1)	184 (42.2)	50 (39.7)	141 (40.3)
Ethnic Background^a^					
Dutch	500 (47.5)	55 (50.0)	221 (49.1)	74 (55.6)	150 (41.8)
Western Non-Dutch	85 (8.1)	9 (8.3)	49 (10.9)	5 (3.8)	22 (6.1)
Non-Western Non-Dutch	467 (44.4)	46 (41.8)	180 (40.0)	54 (40.6)	187 (52.1)
Marital status					
Single	27 (2.6)	4 (3.6)	14 (3.1)	5 (3.8)	4 (1.1)
Married/partner	1026 (97.4)	106 (96.4)	436 (96.9)	128 (96.2)	356 (98.9)
Occupation^a^					
Unemployed	326 (31.2)	33 (30.3)	142 (31.8)	41 (31.1)	110 (30.9)
Employed	718 (68.8)	76 (69.7)	305 (68.2)	91 (68.9)	246 (69.1)
Enabling variables					
Deprived area^a^					
No	835 (79.4)	91 (82.7)	372 (82.9)	110 (82.7)	262 (72.8)
Yes	217 (20.6)	19 (17.3)	77 (17.1)	23 (17.3)	98 (27.2)
Team					
1	530 (50.3)	64 (58.2)	224 (49.8)	71 (53.4)	171 (47.5)
2	523 (49.7)	46 (41.8)	226 (50.2)	62 (46.6)	189 (52.5)
Year					
2015	510 (48.4)	53 (48.2)	224 (49.8)	51 (38.3)	182 (50.6)
2016	543 (51.6)	57 (51.8)	226 (50.2)	82 (61.7)	178 (49.4)
Need variables					
Consultations telephone					
Mean (sd)	2.8 (3.2)	4.7 (4.5)	2.1 (2.4)	4.8 (4.3)	2.3 (2.4)
Parity					
Nulliparous	509 (48.3)	59 (53.6)	222 (49.3)	66 (49.6)	162 (45.0)
Multiparous	544 (51.7)	51 (46.4)	228 (50.7)	67 (50.4)	198 (55.0)
Start of pregnancy^a^					
Spontaneous	989 (94.9)	104 (96.3)	429 (96.4)	117 (88.6)	339 (95.0)
Assisted	53 (5.1)	4 (3.7)	16 (3.6)	15 (11.4)	18 (5.0)
Duration of getting pregnant in months^a^					
mean (sd)	5.2 (10.2)	5.2 (10.1)	4.1 (7.9)	6.7 (13.0)	5.9 (11.2)
Attendance general practitioner					
No	911 (86.5)	96 (87.3)	400 (88.9)	106 (79.7)	309 (85.8)
Yes	142 (13.5)	14 (12.7)	50 (11.1)	27 (20.3)	51 (14.2)
Attendance Specialist					
No	757 (71.9)	82 (74.5)	342 (76.0)	81 (60.9)	252 (70.0)
Yes	296 (28.1)	28 (25.5)	108 (24.0)	52 (39.1)	108 (30.0)
Consultation Obstetrician^a^					
No	478 (45.5)	22 (20.0)	217 (48.3)	36 (27.1)	203 (56.7)
Yes	572 (54.5)	88 (80.0)	232 (51.7)	97 (72.9)	155 (43.3)
Psychosocial problems^a^					
No	749 (71.3)	77 (70.0)	333 (74.5)	72 (54.1)	267 (74.2)
Yes in the past	211 (20.1)	25 (22.7)	87 (19.5)	33 (24.8)	66 (18.3)
Yes at present	90 (8.6)	8 (7.3)	27 (6.0)	28 (21.1)	27 (7.5)
POP consultation					
No	1018 (96.7)	104 (94.5)	443 (98.4)	121 (91.0)	350 (97.2)
Yes	35 (3.3)	6 (5.5)	7 (1.6)	12 (9.0)	10 (2.8)
Sexual or domestic violence^a^					
No	915 (87.1)	91 (82.7)	407 (90.6)	100 (75.2)	317 (88.1)
Yes	136 (12.9)	18 (16.5)	42 (9.4)	33 (24.8)	43 (11.9)
Prescribed Drugs^a^					
No	955 (90.9)	101 (91.8)	420 (93.5)	114 (85.7)	320 (89.1)
Yes	96 (9.1)	9 (8.2)	29 (6.5)	19 (14.3)	39 (10.9)
Number of ultrasounds					
Mean (sd)	3.9 (1.4)	4.1 (1.3)	3.9 (1.4)	4.2 (1.5)	3.7 (1.5)
Health behaviours variables					
Smoking^a^					
No	806 (76.7)	77 (70.0)	363 (80.8)	88 (66.2)	278 (77.4)
Yes	245 (23.3)	33 (30.0)	86 (19.2)	45 (33.8)	81 (22.6)
Alcohol use^a^					
No	1006 (95.7)	101 (91.8)	434 (96.7)	122 (91.7)	349 (97.2)
Yes	45 (4.3)	9 (8.2)	15 (3.3)	11 (8.3)	10 (2.8)
Drugs addiction^a^					
No	1028 (97.8)	109 (99.1)	441 (98.2)	124 (93.2)	354 (98.6)
Yes	23 (2.2)	1 (0.9)	8 (1.8)	9 (6.8)	5 (1.4)
BMI^a^					
Normal weight	656 (62.5)	80 (72.7)	315 (70.5)	64 (48.1)	197 (54.9)
Overweight	256 (24.4)	24 (21.8)	93 (20.8)	46 (34.6)	93 (25.9)
Obese	137 (13.1)	6 (5.5)	39 (8.7)	23 (17.3)	69 (19.2)

^a^sample size varies due to missing data; valid percentages are shown; *POP* Psychiatrist-Obstetrician Pediatricianclinic; education: low (primary school and some vocational training), intermediate (secondary school and completed vocational training), and high (college and/or university)

The underlying reasons for more than the recommended visits in the midwifery practice in the 243 frequent attenders, independent of referral, are shown in Table 2. The most reported reasons were being worried/having vague complaints (44%), followed by abdominal pain (15%), medical problems (13%), psychosocial problems, e.g. behavioral, emotional and/or relational problems (12%) and experiencing high pregnancy burden (8%).

**Table 2:** Underlying reasons for frequent attendance (*N* = 243)

Reason	N (%)
Worried/vague complaints	106 (43.6)
Abdominal complaints without medical explanation	37 (15.2)
Psychosocial problems	29 (11.9)
High experienced pregnancy load	20 (8.2)
Hypertension complaints	11 (4.5)
Previous miscarriage	11 (4.5)
Long-term children wish	8 (3.3)
Blood loss first trimester	7 (2.9)
Blood loss second trimester	4 (1.6)
Nausea	4 (1.6)
Growth retardation	1 (0.4)
Hemoglobin pathology	1 (0.4)
A fetus with a congenital anomaly	1 (0.4)
Combination of reasons	3 (1.2)
Total	243 (100)

Table 3 shows reasons for consultation with an obstetrician and reasons for referral to an obstetrician. More than half of the study population, 572/1053 (55%), had at least one consultation with an obstetrician, ranging from one to seven; the mean of the number of consultations for frequent attenders and adequate users was 2.7 (SD 2.97) and 1.9 (SD 4.83), respectively. Of the whole study population, 493/1053 (47%) were referred to an obstetrician. Half of the women referred, 252/493 (51%) had at least one prior consultation with an obstetrician. Frequent attenders were more likely to be referred compared to adequate users (respectively 54.7 and 44.4%; *p = 0*.005). Women of non-western non-Dutch origin were more likely to be referred compared to women of Dutch or women of western non-Dutch origin (respectively 51.6, 44.8, and 31.8%; *p* = 0.002). Women living in a deprived area were more likely to be referred, compared to women not living in a deprived area (respectively 55.8 and 44.6%; *p* = 0.003).

**Table 3:** Reasons for referral and consultation obstetrician among the entire study population (*N* = 1053)

Reasons	Referral *N* = 493 (46.8%)	Consultation *N* = 574 (54.5%)
Glucose intolerance	138 (27.9)	
Pregnancy-induced hypertension	70 (14.1)	48 (8.4)
Previous C-section	48 (9.7)	22 (3.8)
Postterme (≥42 weeks)/request for induction	30 (6.1)	35 (6.1)
Breech or other malpresentation	27 (5.5)	17 (3.0)
Less fetal movements	23 (4.6)	81 (14.1)
Intercurrent disease	18 (3.6)	
Small for gestational age	17 (3.4)	
Premature birth	15 (3)	
Suspicion of premature birth		20 (3.5)
Congenital anomaly	12 (2.4)	13 (2.3)
Large for gestational age	11 (2.2)	
Anaemia	11 (2.2)	36 (6.3)
Previous small for gestational age	9 (1.8)	
Placenta problems	6 (1.2)	
Hydramnion	6 (1.2)	
Uterus anomaly/cyst/myomas	5 (1)	
Stillbirth	4 (.8)	
Hyperemesis	3 (.6)	
Fetal distress	2 (.4)	16 (2.8)
Blood loss	2 (.4)	39 (6.8)
Psychiatric diseases	1 (.2)	
Psychiatric problems (POP)		12 (2.1)
Twin pregnancy	1 (.2)	
Abdominal pains		74 (12.9)
Fetal growth control		68 (11.8)
Other, such as cholestasis, maternal heart defects, rheumatism, gastric bypass, pelvic pain	34 (6.9)	
Other, such as pelvic pain, GBS, clotting disorder, vague complaints		93 (19.9)

The final logistic regression model on the entire study population included enabling, need, and health behaviors variables, but no predisposing variables. Women who started their prenatal care in 2016, with an assisted start of pregnancy, a consultation with an obstetrician, a history of sexual violence, more than average ultrasounds, and were overweight had higher odds of frequent attendance, whereas women living in a deprived area had lower odds of frequent attendance (Table 4).

**Table 4:** Unadjusted and adjusted associations of various characteristics of women with adequate plus use of care, and split by subsequent referral in the study practice

	Study population		Not referred		Referred	
	Unadjusted OR (CI) *n* ≤ 1053^**a**^	Adjusted OR (CI) *n* = 937	Unadjusted OR (CI) *n* ≤ 560^**a**^	Adjusted OR (CI) *n* = 502	Unadjusted OR (CI) *n* ≤ 493^**a**^	Adjusted OR (CI) *n* = 435
Predisposing variables						
Age	0.98 (0.95–1.01)		0.98 (0.94–1.02)		0.97 (0.93–1.01)	0.95 (0.90–1.00)
Level of Education^a^						
Low	1.00 (ref.)		1.00 (ref.)		1.00 (ref.)	
Middle	1.13 (0.70–1.83)		1.66 (0.74–3.71)		0.91 (0.49–1.69)	
High	1.06 (0.65–1.73)		1.47 (0.65–3.31)		0.91 (0.48–1.71)	
Ethnic Background^a^						
Dutch	1.00 (ref.)		1.00 (ref.)		1.00 (ref.)	
Western Non-Dutch	0.57 (0.31–1.04)		0.74 (0.34–1.59)		0.46 (0.16–1.26)	
Non-Western Non-Dutch	0.78 (0.58–1.01)		1.03 (0.66–1.59)		0.58 (0.38–0.88)	
Occupation^a^						
Unemployed	1.00 (ref.)		1.00 (ref.)		1.00 (ref.)	
Employed	1.03 (0.76–1.41)		1.07 (0.68–1.69)		0.99 (0.64–1.52)	
Enabling variables						
Deprived area^a^						
No	1.00 (ref.)	1.00 (ref.)	1.00 (ref.)		1.00 (ref.)	1.00 (ref.)
Yes	0.76 (0.52–1.10)	0.64 (0.42–0.99)	1.01 (0.58–1.75)		0.55 (0.33–0.92)	0.50 (0.27–0.92)
Team						
1	1.00 (ref.)		1.00 (ref.)		1.00 (ref.)	
2	0.76 (0.57–1.02)		0.71 (0.47–1.09)		0.79 (0.53–1.17)	
Year						
2015	1.00 (ref.)	1.00 (ref.)	1.00 (ref.)		1.00 (ref.)	1.00 (ref.)
2016	1.34 (1.01–1.79)	1.56 (1.12–2.18)	1.07 (0.70–1.62)		1.64 (1.09–2.46)	2.42 (1.45–4.05)
Need variables						
Parity						
Nulliparous	1.00 (ref.)		1.00 (ref.)		1.00 (ref.)	
Multiparous	0.86 (0.73–1.03)		0.87 (0.66–1.14)		0.83 (0.66–1.05)	
Start of pregnancy^a^						
Spontaneous	1.00 (ref.)	1.00 (ref.)	1.00 (ref.)		1.00 (ref.)	
Assisted	1.56 (1.14–2.15)	1.49 (1.04–2.13)	1.32 (0.73–2.37)		1.60 (1.08–2.36)	
Duration of getting pregnant^a^	1.01 (1.00–1.02)		1.01 (0.99–1.04)		1.00 (0.98–1.02)	
Attendance general practitioner						
No	1.00 (ref.)		1.00 (ref.)		1.00 (ref.)	
Yes	1.43 (0.96–2.12)		1.17 (0.62–2.20)		1.54 (0.92–2.58)	
Attendance Specialist						
No	1.00 (ref.)		1.00 (ref.)		1.00 (ref.)	
Yes	1.35 (0.99–1.84)		1.08 (0.67–1.75)		1.49 (0.98–2.26)	
Consultation Obstetrician^a^						
No	1.00 (ref.)	1.00 (ref.)	1.00 (ref.)	1.00 (ref.)	1.00 (ref.)	1.00 (ref.)
Yes	3.46 (2.50–4.79)	3.39 (2.37–4.85)	3.74 (2.26–6.18)	3.99 (2.35–6.77)	3.52 (2.28–5.45)	2.75 (1.66–4.57)
Psychosocial problems^a^	1.00 (ref.)		1.00 (ref.)		1.00 (ref.)	1.00 (ref.)
No	1.53 (1.07–2.17)		1.24 (0.75–2.07)		1.85 (1.13–3.03)	1.85 (1.02–3.35)
Yes in the past	2.69 (1.70–4.25)		1.28 (0.56–2.93)		3.85 (2.13–6.93)	2.99 (1.43–6.25)
Yes at present						
Sexual or domestic violence^a^						
No	1.00 (ref.)	1.00 (ref.)	1.00 (ref.)	1.00 (ref.)	1.00 (ref.)	
Yes	2.27 (1.55–3.33)	2.25 (1.44–3.49)	1.92 (1.06–3.48)	2.17 (1.11–4.24)	2.43 (1.46–4.03)	
Prescribed Drugs^a^						
No	1.00 (ref.)		1.00 (ref.)		1.00 (ref.)	
Yes	1.42 (0.89–2.26)		1.29 (0.59–2.81)		1.36 (0.75–2.46)	
Number of ultrasounds						
(mean)	1.18 (1.07–1.31)	1.18 (1.05–1.33)	1.09 (0.94–1.27)		1.27 (1.11–1.46)	1.38 (1.15–1.65)
Health behaviour variables						
Smoking^a^						
No	1.00 (ref.)	1.00 (ref.)	1.00 (ref.)		1.00 (ref.)	
Yes	1.81 (1.32–2.50)	1.59 (1.10–2.30)	1.81 (1.13–2.90)		1.75 (1.13–2.71)	
BMI^a^						
Normal weight	1.00 (ref.)	1.00 (ref.)	1.00 (ref.)		1.00 (ref.)	1.00 (ref.)
Overweight	1.34 (0.93–1.86)	1.51 (1.04–2.18)	1.02 (0.61–1.70)		1.52 (0.96–2.39)	1.88 (1.09–3.24)
Obese	0.96 (0.61–1.50)	0.80 (0.47–1.35)	0.61 (0.25–1.48)		1.02 (0.59–1.77)	0.79 (0.39–1.58)

^a^sample size varies due to missing data; *OR* odds ratio, *CI* confidence intervals 95%; education: low (primary school and some vocational training), intermediate (secondary school and completed vocational training), and high (college and/or university)

Analyses showed referral to an obstetrician as an effect modifier, therefore, we split the results by referral (Table 4).

### Results of non-referred pregnant women

The characteristics of the non-referred population (frequent attenders and adequate users) are shown in Table 1, columns 3 and 4.

The final logistic regression model on the subset of non-referred participants included only need variables: consultation with an obstetrician and a history of sexual violence. Women with one or more consultations with an obstetrician and women with a history of sexual violence had higher odds of frequent attendance (Table 4, columns 4 and 5).

### Results of referred pregnant women

The characteristics of the referred population (frequent attenders and adequate users) are shown in Table 1, columns 5 and 6.

The final logistic regression model on the subset of referred participants included enabling, need, and health behavior variables: deprived area, year, consultation with an obstetrician, psychosocial problems, number of ultrasounds, and BMI (Table 4, columns 6 and 7). Women living in a deprived area had lower odds of frequent attendance, whereas women who started prenatal care in 2016, with one or more consultations with an obstetrician, psychosocial problems in the past or present, who had more than average ultrasounds, and were overweight had higher odds of frequent attendance.

## Discussion

We assessed the prevalence, determinants and underlying causes of frequent attenders of prenatal care by referred and non-referred women in a large Dutch midwifery practice. This exploratory study showed that almost one in four pregnant women received adequate plus care and were considered frequent attenders. The strongest associations of frequent attendance in both the non-referred and the referred participants were consultation with an obstetrician. In the non-referred group a history of sexual violence, and in the referred group psychosocial problems showed the strongest associations. The underlying causes of frequent attendance were mainly due to worries and/or vague complaints and abdominal pain.

In our study, 23% of the study population meet the criteria and were considered frequent attendees and accounted for a significant number of prenatal visits. Our findings are in line with a large retrospective study in the United States that reported a frequent attendance rate of 30% among low-risk pregnant women [14]. Contrary, a Dutch national study reported a prevalence of frequent attendance of 13% between 2009 and 2011 in primary midwifery care [3]. The higher frequent attendance rate in the current study could be explained by several interventions that were introduced since 2011 to improve perinatal care. These interventions were introduced because of a relatively high perinatal mortality rate in the Netherlands compared to other European countries and were aimed at increasing awareness for psychosocial problems, e.g. implementation of the Psychiatrist-Obstetrician-Pediatrician -consultations, and mainly targeted at women living in deprived areas and women with a non-western non-Dutch background [18–20].

A majority of frequent attenders had complaints related to psycho-social problems, a broader definition including everything that affects the functioning of woman’s daily life such as worries or having vague complaints [21], one of the perceived needs according to Andersen’s model; medical reasons were less identified. This observation is in accordance with previous studies in general practice [8, 22]. Pregnancy-related anxiety, defined as worries and fears related to the health of the child, giving birth, parenting and/or caring for the child, is an important psychological factor [23, 24]. Most pregnant women report pregnancy-related anxiety, which may be in the continuum of normality since it is an indicator of an important motherhood task of protecting and caring for the unborn baby [24]. Nevertheless, higher levels of pregnancy-related anxiety is a known risk factor for adverse perinatal outcomes [25–28]. Preterm birth, low birth weight, perinatal mortality, and lower Apgar scores are also related to frequent attendance [11–13].

Even though medical reasons were less reported as an underlying cause, frequent attenders did often have consultations with an obstetrician, one of the evaluated needs according to Andersen’s model. These consultations were mainly because of medical reasons such as decrease in fetal growth, less fetal movements, and blood loss. The beforementioned symptoms are related to fetal distress and obstetricians could more carefully monitor the condition of the fetus for the best possible outcomes.

Finally, we found a strong association between frequent attendance and a history of sexual violence, one of the perceived needs according to Andersen’s model. Sexual and domestic violence is an important problem worldwide. The prevalence of sexual violence under the age of 18 is over 20%, with even a higher prevalence across all ages [29, 30]. The reported prevalence of sexual violence during pregnancy is 17% while only a third of the women disclose the sexual violence to their caregiver [31, 32]. Compared to women without a history of sexual violence, women exposed to sexual violence are more likely to fear child birthing, have negative birth experiences and adverse perinatal outcomes [31, 33]. Building trusting relationships with women is of importance to improve care and to facilitate births in a sensitive way so that re-enactment of abuse will be minimized [1]. In 2017, the #metoo movement started a public confession about the sexual violence of famous and non-famous women. As a subsequent result, a public debate on this subject started in many countries. The #MeToo movement is of great importance to provide a safe and supportive environment where sexual violence could be openly discussed, including in maternity/prenatal care. As only about a third of pregnant women reveal sexual violence to their caregiver, we expect an underreporting of women who experienced sexual violence. Therefore this subject should be discussed on a regular basis throughout the pregnancy. We also recommend being alert to physical complaints such as chronic pelvic pain, as these could indicate a history of sexual violence [34].

To the best of our knowledge, this is the first study to explore the phenomenon of frequent attendance in primary midwifery care. The dataset was robust with detailed and unique information; de-identified and anonymized data were extracted from the digital midwifery database including medical charts, containing information concerning sexual violence and psychosocial problems. These facts are not available in national registrations of birth outcomes. The study cohort was similar to the Dutch national pregnant population women with respect to average age (29.2 years versus 31.1 years), and proportion of primipara (29.2 years versus 31.1 years) [17]. However, the proportion of non-Dutch women in our study was significantly higher (53% versus 26%); this could explain the higher prevalence of referral compared to the national pregnant population (47 and 35%) [17]. Furthermore, our sample size was large enough to examine the distinction between referred and non-referred pregnant women and showed that these two groups had different medical and non-medical problems that needed more care provided by the midwives.

The performance statistics of the regression model on the subset of referred participants indicate that this model could explain well the occurrence of frequent attendance and that the results could be generalized to the underlying population of pregnant women in prenatal care. In contrast, the performance statistics of the regression models on the entire study population and the subset of non-referred participants indicated that these models could not completely explain the occurrence of frequent attendance and could not be generalized to the underlying population of pregnant women in prenatal care. Different models might be obtained with a larger and different study population, adjusting for several relevant factors that were not included in this study [35, 36]; e.g. mental health was not included in our study, whereas women with a mental disability have more prenatal visits compared to women without [37].

Our research recommendation is to repeat this study in a larger database that includes multiple practices to make the results more generalizable. In addition, we recommend exploring, through qualitative research, women’s underlying reasons and perceptions regarding the use of prenatal health care. This would add information for policymakers in order to adjust prenatal guidelines to the needs of pregnant women.

## Conclusions

In our exploratory study of low-risk women, frequent attendance was observed in almost one in four pregnant women mainly because of non-medical reasons. Associations with frequent attendance differ between non-referred and referred pregnant women. Non-referred frequent attenders had relatively more a history of sexual violence, whereas referred frequent attenders had relatively more often psychosocial problems.

### Implications for practice

To provide the best possible care, a trustful midwife-woman relationship is needed for women to share more details in case of vague complaints or worries to identify their implicit needs. Furthermore, the presence of sexual violence should be explored at least twice during pregnancy, because only a third of women disclose a history of sexual violence during pregnancy. Such information can be used by midwives to attune the prenatal care to the woman’s specific needs. This implies that (student-)midwives have to learn to be flexible with the guidelines about the prenatal visits schedule and have to decide together with every individual pregnant woman when and how prenatal care is provided.

## Appendix

Kotelchuck Index-Revised. Index for assessment of the adequacy of prenatal care utilization in the Dutch primary midwifery care context.

This index, based on the Kotelchuck Index, is a validated tool for measuring the level of care utilization, is based on the number of face-to-face visits with the midwife, adjusted for gestation and onset of care and aligned with the number of recommended face-to-face prenatal visits by the KNOV. Utilization of care was defined as inadequate (less than 50% of the recommended visits), intermediate (50–79%), adequate (80–109%) and adequate plus (110% or more).

**Table 5:** Index for assessment of the adequacy of prenatal care utilization in the Dutch primary midwifery care context [3]

Duration of gestation	Initiation of care	Number of visits	Kotelchuck Index^b^
0 - 11+6	≤ 11+6	≥ 3	4
		1-2	3
			2
		0	1
12 - 26+6 Ideally 3.75 visits^a^	≤ 11+6	≥ 6	4
		3-5	3
		2	2
		≤ 1	1
	≥ 12+0		1
27 - 36+6 Ideally 7.5 visits^a^		≥ 10	4
		**6-9**	3
		4-5	2
		≤ 3	1
	≥ 12+0		1
37+0 - 37+6 Ideally 11 visits^a^	≤ 11+6	≥ 13	4
		10-12	3
		6-9	2
		≤5	1
	≥ 12+0		1
38+0 - 38+6 Ideally 12 visits^a^	≤ 11+6	≥ 14	4
		10-13	3
		6-9	2
		≤5	1
	≥ 12+0		1
39+0 - 39+6 Ideally 13 visits^a^	≤ 11+6	≥ 15	4
		11-14	3
		7-10	2
		≤ 6	1
	≥ 12+0		1
40+0 - 40+6 Ideally 14 visits^a^	≤ 11+6	≥ 16	4
		12-15	3
		7-11	2
		≤ 6	1
	≥ 12+0		1
41+0 - 41+6 Ideally 15 visits^a^	≤ 11+6	≥ 17	4
		**12-16***	3
		**8-11**	2
		≤ 7	1
	≥ 12+0		1

^a^According to the guidelines of the Royal Dutch Organization of Midwives

^b^Kotelchuck Index:

1. Inadequate (received less than 50% of expected visits)

2. Intermediate (50%-79%)

3. Adequate (80%-109%)

4. Adequate Plus (110% and more)

## Abbreviations

KNOV: Koninklijke Organisatie van Verloskundigen - the Royal Dutch Organisation of Midwives
POP: Psychiatrist-Obstetrician-Pediatrician-clinic
